# Effect of technologist and patient attributes on centering for body CT examinations: Influence of cultural and ethnic factors

**DOI:** 10.1371/journal.pone.0273227

**Published:** 2022-08-19

**Authors:** Antar Aly, Shadi Ebrahimian, Mohammed H. Kharita, Mahmoud Heidous, Mohammad Zaya Ashruf, Davendra Kumar, Mannudeep K. Kalra, Huda Mohd Al Naemi

**Affiliations:** 1 Hamad Medical Corporation, Doha, Qatar; 2 Department of Radiology, Massachusetts General Hospital, Harvard Medical School, Boston, Massachusetts, United States of America; Universiti Teknologi Malaysia - Main Campus Skudai: Universiti Teknologi Malaysia, MALAYSIA

## Abstract

There are no published data on the effect of patient and technologist gender and ethnicity attributes on off-centering in CT. Therefore, we assessed the impact of patient and technologist variations on off-centering patients undergoing body CT. With institutional review board approval, our retrospective study included 1000 consecutive adult patients (age ranged 22–96 years; 756 males: 244 females) who underwent chest or abdomen CT examinations. We recorded patient (age, gender, nationality, body weight, height,), technologist gender, and scan-related (scanner vendor, body region imaged, scan length, CT dose index volume, dose length product) information. Lateral and anteroposterior (AP) diameters were recorded to calculate effective diameter and size-specific dose estimate (SSDE). Off-centering represented the distance between the anterior-posterior centers of the scan field of view and the patient at the level of carina (for chest CT) and iliac crest (for abdomen CT). About 76% of the patients (760/1000) were off-centered with greater off-centering for chest (22 mm) than for abdomen (15 mm). Although ethnicity or patient gender was not a significant determinant of off-centering, technologist-patient gender mismatch was associated with a significantly greater frequency of off-centering (p<0.001). Off-centering below the gantry isocenter was twice as common as off-centering above the gantry isocenter (p<0.001). The latter occurred more frequently in larger patients and was associated with higher radiation doses than those centered below the isocenter (p<0.001). Technologists’ years of experience and patient factors profoundly affect the presence and extent of off-centering for both chest and abdomen CTs. Larger patients are more often off-centered than smaller patients.

## Introduction

Most modern CT scanners are equipped with automatic tube current modulation techniques, which modulate tube current based on the patient’s attenuation profile and the specified image quality metric [1, 2]. In addition, some newer scanners use automatic potential selection techniques that select tube potential based on the type of CT examination patient’s attenuation profile [3, 4]. The patient’s attenuation profile information is estimated from the localizers or planning radiographs acquired before actual scanning. The ability to accurately determine patients’ attenuation profiles from the planning radiographs depends on patients centering in the CT gantry isocenter. Patient off-centering can lead to erroneous estimation of patient size and attenuation, leading to the suboptimal selection of tube current and tube potential required to obtain Images with the desired image quality and radiation dose.

Beyond the automatic tube current modulation and tube potential selection techniques, multidetector-row CT scanners have beam shaping or bow tie filters which help homogenize image quality across the image cross-section and reduce surface radiation dose [5]. In patients with optimal centering in gantry isocenter, these beam shaping filters reduce peripheral radiation dose while allowing most X-ray photons to travel through the central part of the patient’s cross-section. However, in off-centered patients, the central thicker part of the patient’s cross-section may receive less radiation dose (resulting in more artifacts and image noise), while the peripheral thinner portions can receive higher radiation dose (lower image noise). Off-centered patients in scanners with beam shaping filters have an inhomogeneous distribution of noise in different anatomy parts. In recognition of its importance, some newest CT scanners employ 3D camera and artificial intelligence to automate patient centering; their availability on most scanners is limited [6].

Several prior studies have reported the frequency and effect of patient off-centering in the CT gantry isocenter [7–9]. However, to our best knowledge, prevalence off-centering on gender mismatch between the patient and CT technologist in the context of a conservative Middle East population has not been reported. Furthermore, substantial cultural differences and norms in different parts of the world support a need to conduct such evaluation and undertake targeted strategies to address issues with patient off-centering. Therefore, the purpose of our study was to assess the effect of variations in patient and technologist attributes on off-centering of patients undergoing body CT examinations in the Middle East.

## Methods and materials

This study was exempted under Ministry of Public Health (MOPH) guidelines, research involving the collection or study of existing: data, documents, records and the information is recorded by the investigator in such a manner that subjects cannot be identified, directly or through identifiers linked to the subjects. Under the exempt status, the need for obtaining informed consent is waived. Our observational study received institutional review board (IRB) approval from the human research committee of the HMC Medical Research Centre (MRC) (Protocol ID: MRC-01-21-477). A study coauthor (MKK) received research grants from Siemens Healthineers, Riverain Tech, and Coreline Inc. for unrelated projects. None of the authors have any pertinent financial disclosures related to the submitted work.

### Patients and CT technologists

The study included 1000 consecutive adult patients (age range 22–96 years; 756 males: 244 female) who underwent standard of care chest or abdomen CT examinations at Al-Wakra Hospital, a tertiary care hospital-based in Al-Wakra, Qatar from 1st January 2021 to 1st May 2021. All CT examinations represented the standard of care imaging as part of patients’ clinical care, regardless of clinical indications. Most common indications for chest and abdomen CT examinations were cancer staging, treatment response, and surveillance.

For each patient, we recorded the following information at the time of scanning: date of CT examination, body part imaged, patient age, gender, body weight, and height. Body mass index was estimated as a surrogate for patient size. In addition, the nationality of each patient was recorded and classified into one of the geographic regions for analysis: the Middle East, Asia (not including the Middle East), Africa, Europe, and Americas (North and South American patients were combined together due to small sample size). There were no patients from Australia.

In addition to the patient information, we recorded CT technologists’ gender and years in service as CT technologists.

### CT

All patients underwent chest or abdominal CT on one of the two multidetector-row, single-source CT scanners: 128-detector-row (Philips iCT, Philips Healthcare, Eindhoven, The Netherlands) or 320-detector-row (Canon Aquilion, Canon Medical Systems Corporation, Tochigi, Japan) scanners. All patients were scanned in a supine position and with a single-breath-hold. Per standard of care, scan parameters for all CT examinations included: 100–120 kV, vendor-specific automatic tube current modulation techniques, scanner-selected pitch or 0.9:1 beam pitch, and 0.5 (chest) or 0.8 (abdomen) second gantry rotation times.

Apart from the standard of care skin-in-skin reconstructed images for routine chest or abdomen CT, technologists reconstructed a single transverse CT image with full 50 cm field of view (FOV) at the level of tracheal bifurcation (carina) for each chest CT exam within 1 cm of the iliac crest for each abdomen CT. These full FOV images were transferred to PACS for estimating patient centering.

### Off centering assessment

To estimate off-centering, one of the two CT technologists first drew a line connecting the anterior and posterior margin of the full FOV and determined the gantry isocenter at the midpoint of this line. Patient’s center corresponded to the midpoint of a straight line connecting the anterior and posterior surface of the patient. The distance between the gantry isocenter and patient center conveyed information on patient centering in the scanner. Distance less than or equal to 0.5 cm was deemed optimal (patient adequately centered), while distance greater than 0.5 cm suggested off-centering. The off-centering direction was classified as gantry table too high (patient was centered above the gantry isocenter) or too low (patient was below the gantry isocenter). In addition to centering, scan start and end locations were recorded to estimate scan length for each CT. body part imaged, patient age, gender, body weight, and height. Body mass index was estimated as a surrogate for patient size. In addition, the nationality of each patient was recorded and classified into one of the geographic regions for analysis: the Middle East, Asia (not including the Middle East), Africa, Europe, and North America. There were no patients from South America and Australia.

In addition to the patient information, we recorded CT technologists’ gender and years in service as CT technologists.

### Radiation dose

We recorded volume CT dose index (CTDIvol in milli-Gray or mGy) and dose length product (DLP in in milli-Gray.cm or mGy.cm) from the dose information page for each CT examination. The dose data were collected to determine how off-centering affects CTDLvol and DLP in patients with similar size and scan protocol. From the full FOV image, each patient’s anterior-posterior and lateral diameters were measured to estimate effective diameter (square root of the product of anterior-posterior and lateral diameters). From the look-up table, we obtained the constant which was multiplied with CTDIvol to obtain size-specific dose estimate (SSDE) for each CT [10].

### Statistical analysis

Statistical analysis of the data was performed in Microsoft Excel (Microsoft Inc. Redmond, Washington) and SPSS Statistical Software (IBM Inc. Chicago, IL). Since the data were not normally distributed, we estimated the median and interquartile range (IQR) of patient weights, body mass index, effective diameters, off-centering distance, CTDIvol, and DLP. We assessed the differences in off-centering and radiation doses for patients of different gender, age group, geographic origin, as well as the body region and scanner. Chi-squared and Kruskal-Wallis omnibus tests (which include the post-hoc tests for pairwise testing of variables within the SPSS software) were used for statistical analysis. P-values < 0.05 was considered as the significance level.

## Results

### Off-centering frequency and extent

Of the 1000 patients included in our study, more than three-fourths of the patients (76%; 760/1000) were off-centered, and 24% were centered correctly (240/1000). The median off-centering distance for chest CT exams (22 mm, IQR 19) was more significant than for abdominal CT (15 mm, IQR 13) (p = 0.003). In addition, off-centering below the gantry isocenter (55.9%; 559/1000) was more frequent than above the gantry isocenter (20.1%, 201/1000). Table 1 summarizes differences in patient and radiation doses with and without off-centering for chest and abdomen CT examinations. Off-centering was slightly but statistically more common in chest CT (92/114; 80.7%) than in abdomen CT (668/886; 75.4%) (p<0.001). There was no difference in scan lengths between centered and off-centered patients (p = 0.102).

**Table 1:** Summary of patient characteristics and radiation doses for chest and abdomen CT examinations performed with optimal and suboptimal patient centering.

*Variables*	*Chest CT*		*Abdomen CT*	
	*Centered*	*Off-centered*	*Centered*	*Off-centered*
*Age (years)*	46 (26)	50 (23)	39 (20)	37 (20)
*BMI (kg/m* ^*2*^ *)*	25.8 (8.6)	27.2 (5.3)	26.8 (5.8)	26.6 (7.4)
*Effective diameter (mm)*	297 (36)	295 (37)	288 (46)	285 (62)
*Scan length (mm)*	322 (63)	298 (77)	408 (64)	417 (57)
*Centering distance (mm)*	3.0 (1.8)	22.0 (19.4)	3.0 (2.0)	15.0 (13.0)
*CTDIvol (mGy)*	8.6 (4.3)	8.5 (3.9)	10.2 (4.3)	9.8 (5.5)
*SSDE (mGy)*	10.5 (4.7)	10.7 (3.7)	13.1 (4.1)	12.9 (4.3)
*DLP (mGy*.*cm)*	310.8 (221.5)	319.0 (188.9)	485.8 (221.9)	492.2 (293.6)

All values represent medians (interquartile range).

The distance between patient center and the scanner isocenter was significantly greater for all off-centered chest and abdomen CT examinations (15 mm, IQR 14) than for optimally centered CT (3mm, IQR 2) (p<0.001). For all CT examinations performed with and without optimal centering, there were no significant differences in patients’ age (39 vs. 38 years; p = 0.557), gender (p = 0.923), geographic regions (p = 0.876), body mass index (26.8 vs 26.7 kg/m2; p = 0.762), CTDIvol (9.9 vs. 9.6 mGy; p = 0.221), and DLP (477 vs 470 mGy.cm; p = 0.739). Off-centering was common on both vendors’ scanners included in our study (Table 2).

**Table 2:** Summary of patients, radiation doses, scanners, and body regions for CT examinations performed with off-centering below or above the gantry isocenter.

		*Centered*	*Off-centered below gantry isocenter*	*Off-centered above gantry isocenter*	*P-Value*
*Age (Median)*		39 (20)	36 (20)	43 (19)	<0.001
*Gender*	*Female (n = 244)*	58 (23.8%)	128 (52.5%)	58 (23.8%)	0.261
	*Male (n = 756)*	182 (24.1%)	430 (56.9%)	144 (19.0%)	
*Region*	*Africa (n = 181)*	26%	50.3%	23.8%	0.367
	*America (n = 16)*	31.3%	43.8%	25.0%	
	*Asia (n = 509)*	22.4%	60.5%	17.1%	
	*Europe (n = 8)*	25.0%	50.0%	25.0%	
	*Middle East (n = 267)*	25.1%	52.1%	22.8%	
Height (cm)*		169 (12)	168 (10)	169 (13)	0.902
Weight (kg)*		76 (19)	74 (20)	82 (22)	<0.001
*BMI (kg/m* ^*2*^ ) *		26.8 (5.7)	26.0 (6.0)	29.3 (7.1)	<0.001
Effective diameter (cm)*		28.9 (4.4)	27.9 (5.5)	30.5 (6.0)	<0.001
Scan length (mm)*		405 (72)	411 (69)	412 (63)	0.102
Centering distance (mm)*		3.0 (2.0)	16.5 (15.2)	12.0 (11.0)	<0.001
CTDIvol (mGy)*		9.9 (8.0)	9.2 (4.7)	11.1 (6.6)	<0.001
SSDE (mGy)*		12.9 (4.1)	12.4 (3.7)	13.6 (5.6)	0.010
*DLP (mGy*.cm) *		477 (242)	442 (260)	559 (346)	<0.001
*Modality*	*Canon (n = 193)*	61 (31.6%)	65 (33.7%)	67 (34.7%)	<0.001
	*Philips (n = 807)*	179 (22.2%)	493 (61.1%)	135 (16.7%)	
*Organ*	*Abdomen (n = 886)*	218 (24.6%)	476 (53.7%)	192 (21.7%)	<0.001
	*Chest (n = 114)*	22 (19.3%)	82 (71.9%)	10 (8.8%)	

The asterisk (*) represents variables with median values (interquartile range).

### Patient attributes and off-centering

**Fig 1:**
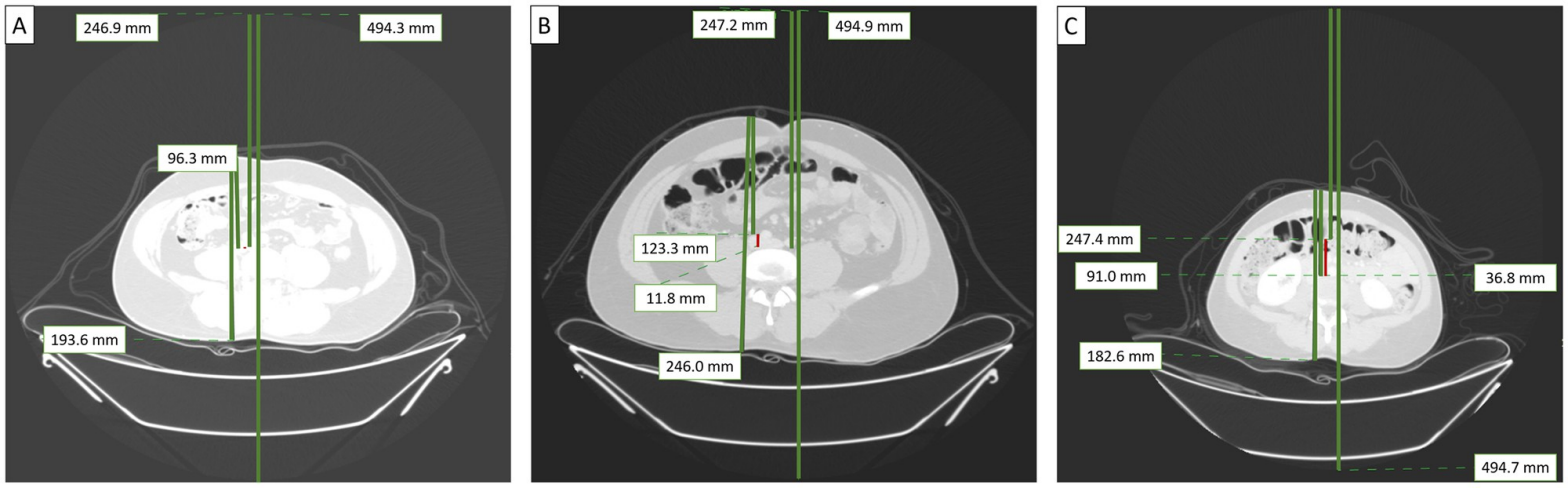
Transverse CT images of three patients reconstructed with full field of view (FOV). Patient A was centered optimally in the CT gantry. Patient B was centered above the gantry isocenter (+11.8 mm) while patient C was centered below the gantry isocenter (–36.8 mm).

Table 2 summarizes patient characteristics and radiation doses in centered patients versus those who were either too low or too high with respect to the gantry isocenter. Fig 1 illustrates chest and abdomen CT examinations performed with good and suboptimal centering. Centering of patients below the gantry isocenter was more common for chest CT and on Philips scanner than for abdomen CT and Canon scanner (both p<0.001). There was no significant variation in off-centering direction or magnitude among patients from different geographic regions (p = 0.367).

**Fig 2:**
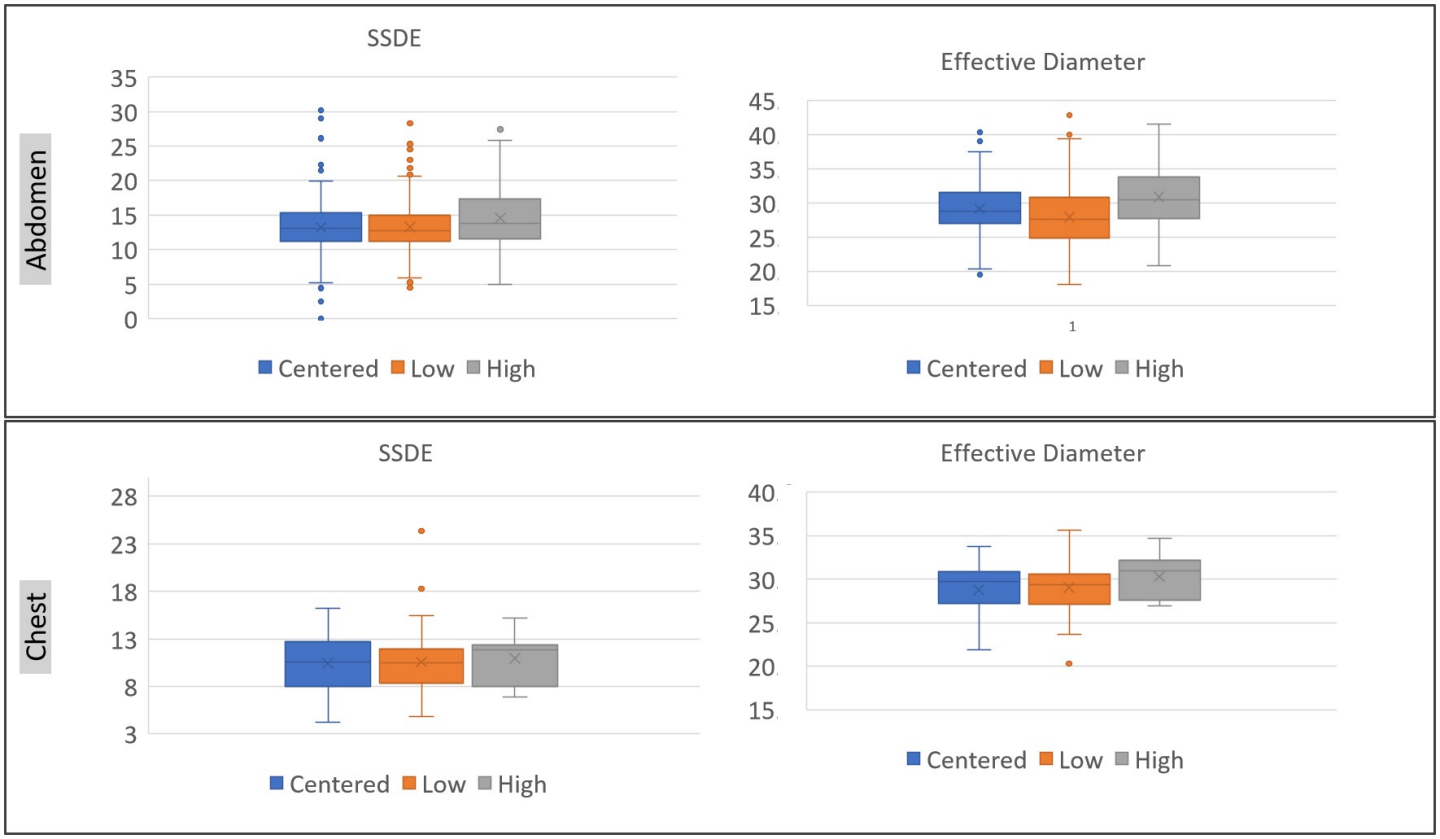
Bar diagrams summarize the distribution of SSDE and effective diameters in chest and abdomen CT for patients scanned with optimal centering versus those centered too low or too high with respect to the gantry isocenter. Note that the patients with high off-centering were larger and received higher radiation dose in contradiction to patients with low off-centering.

Patients centered above the gantry isocenter had greater body weight, BMI, and effective diameter than those centered below the isocenter (Table 2; Fig 2). About one-third of larger patients (BMI ≥30 kg/m2; CTDIvol 15 mGy, IQR 7) were off-centered above the gantry isocenter as compared to 15% (101/671 patients) of non-obese patients (BMI < 30 kg/m2; CTDIvol 9 mGy, IQR 3) (p< 0.001). A similar proportion of men and women were off-centered below or above the gantry isocenter. Patients centered above the gantry isocenter also received higher CTDIvol, DLP, and SSDE than those scanned at low table height (centered below the gantry isocenter) (p<0.01) (Table 2).

### Technologists factors

The proportion of CT examinations with and without off-centering varied significantly across different CT technologists (14–37% versus 63–86%) (p< 0.001). There was also a difference in the extent of off-centering based on technologists’ gender however skewed distribution of technologists’ gender (Table 3) limited statistical comparison. Technologists with greater experience (> 15 years) had a lower rate of off-centering as compared to those with less experience (< 15 years) (p≤ 0.005). Gender mismatch between technologists and patients was associated with a higher frequency of off-centering than CT exams performed with matching technologist and patient genders (p< 0.037). Although not statistically significant (p = 0.074), the median off-centering distance for mismatched patient-technologist gender (12.8 mm, IQR 13 mm) was higher than with matching gender (11.0 mm, IQR 16 mm) for both chest and abdomen CT examinations.

**Table 3:** Summary of technologist factors in centered and off-centered CT examinations.

		*Centered*	*Off-centered below gantry isocenter*	*Off-centered above gantry isocenter*	*P-Value*
*Technologist codes*	*A (n = 135)*	22 (16.3%)	105 (77.8%)	8 (5.9%)	<0.001
	*B (n = 131)*	48 (36.6%)	44 (33.6%)	39 (29.8%)	
	*C (n = 73)*	18 (24.7%)	30 (41.1%)	25 (34.2%)	
	*D (n = 148)*	22 (14.9%)	101 (68.2%)	25 (16.9%)	
	*E (n = 82)*	25 (30.5%)	36 (43.9%)	21 (25.6%)	
	*F (n = 121)*	34 (28.1%)	62 (51.2%)	25 (20.7%)	
	*G (n = 96)*	12 (12.5%)	82 (85.4%)	2 (2.1%)	
	*H (n = 7)*	1 (14.3%)	5 (71.4%)	1 (14.3%)	
	*I (n = 116)*	25 (21.6%)	62 (53.4%)	29 (25.0%)	
	*J (n = 90)*	33 (36.7%)	31 (34.4%)	26 (28.9%)	
*Years of experience*	*5–10 years*	158 (24.8%)	327 (51.3%)	152 (23.9%)	<0.001
	*(n = 637)*				
	*10–15 years*	34 (14.7%)	187 (81.0%)	10 (4.3%)	
	*(n = 231)*				
	*15–20 years (n = 131)*	48 (36.6%)	44 (33.6%)	39 (29.8%)	
*Technologist gender*	*Female*	28 (17.0%)	111 (67.3%)	26 (15.8%)	0.005
	*(n = 165)*				
	*Male*	212 (25.4%)	448 (53.6%)	175 (21.0%)	
	*(n = 835)*				
*Technologist and patient gender*	*Gender match (n = 696)*	180 (25.9%)	385 (55.3%)	131 (18.8%)	0.037
	*Gender mismatch (n = 304)*	60 (19.7%)	173 (56.9%)	71 (23.4%)	

All variables were significantly different across CT performed with and without off-centering.

## Discussion

Like several prior studies, our results document the high frequency of patient off-centering for both chest and abdomen CT examinations [7]. However, we also report vital factors that can help mitigate some causes of off-centering. Patient-technologist gender mismatch, especially in a conservative Arab country, is associated with a higher frequency of off-centering. Contrary to our expectation in the context of the study site, female patients were not off-centered with higher frequency than male patients. Also, chest CT exams were more often off-centered than abdomen CT. This difference may be attributed to breasts in female patients or more complex external anatomy of the chest than the abdomen.

Interestingly, patients centered above the gantry isocenter tended to be larger than those centered below the isocenter. Although most patients were centered below the gantry isocenter in all BMI groups (obese versus non-obese patients) as reported in prior publications [7, 11], a larger proportion of obese (BMI 30–35 kg/m2) and morbidly obese (BMI >35 kg/m2) patients were centered above the gantry isocenter. In contrast, the patients with smaller body habitus were frequently centered below the gantry isocenter. Off-centering above the gantry isocenter for the obese patients is likely co-incidental given the high prevalence of off-centering in most patients regardless of their size or related to errors in estimating centering in larger patients. Besides the higher tube current use for patients with large body sizes, off-centering of obese patients above the gantry isocenter can further increase the radiation dose associated with automatic exposure control techniques due to magnification of projection radiograph at patient centering above the isocenter.

There was no impact of patients’ geographic region of origin on the frequency of off-centering, possibly related to a high prevalence of off-centering regardless of patients’ geographic origins.

There are several clinical implications of our study. Apart from understanding the causes of off-centering from the perspective of a conservative society, our results have led us to initiate a comprehensive mitigation quality improvement program at our site, which we hope to extend across our multi-institutional healthcare enterprise. First, we shared our results with all CT technologists and department leadership to appraise them about the issues with patient centering. Second, we formally educated the CT technologists on the frequency and impact of off-centering in CT. Third, we emphasized the need to center the patient rather than re-center the reconstruction field of view. Fourth, a decision was made to post a note on each CT gantry to remind technologists that their last step before exiting the gantry room for scanning is to turn on the laser markers and check the centering. Fifth, we implemented a form that each technologist must fill to explain the reasons for off-centering. The reasons pertained to complex anatomy (kyphoscoliosis), inability to raise arms over their head, morbid obesity, critical patient with lines/ tubes/life support devices, and "forgot to check patient centering before centering." Sixth, two technologists were assigned to conduct a periodic prospective evaluation of off-centering and reward the best performing technologists. Seventh, need and program to train the support staff of both genders to aid in patient centering in case of patient-technologist gender mismatch. Eighth, we have decided to extend our findings and patient centering strategies to multiple other CT sites affiliated with our healthcare system. Though it is not possible to replace and upgrade to automatic AI-based patient positioning and centering technologies on all scanners, we hope that procurement of such technologies will help address manual errors in off-centering. We believe that our future studies will establish the value of continuous monitoring in mitigating issues related to patient off-centering.

Our study has limitations. Our study was limited to data from a single site and may not generalize across other practices. The data were skewed towards male technologists due to the disproportionately more male than female technologists in our department. There were also more male patients and abdominal CT examinations than female patients and chest CT. With a higher or more symmetric dataset, the study could have yielded different results and conclusions. Still, we did not want to induce non-consecutive or selection bias in our study. Although we withheld information from all technologists about evaluating their centering data, we cannot be sure if some technologists came to know about the audit due to the need to generate an additional CT image in each CT included in our study. Yet, given the similarities across patients’ and technologists’ demographics across many Arabic countries, it is likely that some factors will have similar performance across sites in the region.

Another limitation of our study included the lack of CT head, face, or neck since we wanted to investigate body CT examinations in more challenging regions. We also limited our evaluation to off-centering in the y-axis since prior work suggests that x-axis off-centering is less severe (mean off-centering 0.01 cm), and therefore, less impactful [7, 12]. Some new scanners are equipped with 3D-camera and AI-assisted patient centering capabilities. Since our site does not have scanners with these capabilities, we cannot compare the relative advantage of such techniques that are still limited to some vendors’ most advanced scanners. We also did not the effect and implication of lateral off-centering, and therefore cannot comment on off-centering beyond in the vertical direction. Moreover, we did not use water equivalent diameter for estimating SSDE and instead, relied on measurement of patient dimeters as described in prior publications [6].

Due to unintended skewed distribution of male and female patients and technologists at the study site, the results of our study might not be generalizable at sites with more balanced distribution of patients. However, as stated above, our study included consecutive patients and exams to avoid any selection or temporal biases. Finally, our study did not assess the impact of off-centering on image quality and diagnostic information.

In conclusion, off-centering of patients during body CT examination is up to three times more common than optimal centering, regardless of patient gender, size, ethnicity, scanner type, and technologists’ experience. Gender mismatches between technologists and patients are associated with higher off-centering in a conservative society. Targeted mitigating strategies are required to address the issue of off-centering at such sites.
